# Overlapping exposure to cigarette smoke and particulate matter does not have a direct additive effect on chronic obstructive pulmonary disease

**DOI:** 10.1016/j.pccm.2025.02.006

**Published:** 2025-03-14

**Authors:** Lifeng Yan, Huaqi Guo, Juan Fu, Tianyu Zhou

**Affiliations:** a Department of Respiratory and Critical Care Medicine, Shanghai Ninth People's Hospital, Shanghai Jiao Tong University School of Medicine, Shanghai 200011, China; b The Sidney Kimmel Comprehensive Cancer Center and Department of Oncology, Johns Hopkins University School of Medicine, Baltimore, MD 21287, USA; c The Pancreatic Cancer Precision Medicine Center of Excellence Program, Johns Hopkins University School of Medicine, Baltimore, MD 21287, USA; d The Bloomberg-Kimmel Institute for Cancer Immunotherapy, Johns Hopkins University School of Medicine, Baltimore, MD 21287, USA; e Shanghai Key Laboratory of Tissue Engineering, Shanghai Ninth People's Hospital, Shanghai Jiao Tong University School of Medicine, Shanghai 200011, China

*To the Editor,*

The *Lancet* Commission^1^ and the Global Initiative for Chronic Obstructive Lung Disease (GOLD)^2^ guidelines both proposed a new classification (taxonomy) for chronic obstructive pulmonary disease (COPD). This new taxonomy highlights the heterogeneity of early physiological characteristics, which indicates that recognizing potential hazard factors should be the first and essential step in diagnosing COPD.

The latest *Lancet* Commission and GOLD guidelines equate environmental pollutants with cigarette smoking as an independent risk factor for COPD. The environment in which we live is complex and ever changing. When considering the impacts of cigarette smoke and environmental pollutants individually, we cannot ignore their overlapping effects. Based on our previous study, short-term exposure to particulate matter (PM) has a significantly greater impact on lungs affected by cigarette smoke-related inflammation than on normal lungs.^3^ We speculated that PM might accelerate the onset and progression of cigarette smoke-associated COPD. Thus, we established a 3-month model to observe whether continuous exposure to PM can lead to the premature development of cigarette smoke-associated COPD based on evaluations such as mucus hypersecretion, fibrotic remodeling, and alveolar structure destruction. All experimental operations were authorized by the Institutional Review and Ethics Board of Shanghai Ninth People's Hospital, Shanghai Jiao Tong University School of Medicine (No. SH9H-2023-A883-1).

In our study, 32 eight-week-old male C57BL/6 mice (Shanghai Jie Si Jie Laboratory Animals Co., Ltd., Shanghai, China) were divided randomly into four groups using the random number table method: (1) saline-treated control mice, (2) PM-treated mice, (3) cigarette smoke (CS)-treated mice, and (4) PM–CS-treated mice. According to our previous work,^3^^,^^4^ PM was purchased from Supelco (PEA1906, Merck, Darmstadt, Germany). Male C57BL/6 mice were orotracheally instilled with 30 µL PM (3 mg/kg body weight) or normal saline under anesthesia every three days for 3 months. CS-treated mice and PM–CS-treated mice were exposed to cigarette smoke 30 min twice a day for 3 months. Cigarette smoke of 5 cigarettes (CHIENMEN with filter; British American Tobacco [BAT], Beijing, China) was pumped into a self-designed animal cabin with an oxygen/carbon dioxide concentration sensor, intake valves, and small ventilation openings under negative pressure. After three months of exposure, mice were euthanized using an overdose of isoflurane anesthesia followed by cervical dislocation.

The superior lobe of the right lung was fixed in 4 % paraformaldehyde, and the tissues were embedded in paraffin and cut into 5-µm-thick sections for histopathologic examination. Lung sections were stained with hematoxylin-eosin (HE), periodic acid-Schiff (PAS), and Masson's trichrome for routine morphological analysis. Mucin 5AC (Muc5ac) and α-smooth muscle actin (α-SMA) expression in lung sections was detected by immunohistochemistry (IHC).

Image-Pro Plus 6.0 (Media Cybernetics, Rockville, MD, USA) was used to calculate the mean linear intercept (MLI) and mean alveolar area (MAA) for alveolar enlargement assessment according to previous studies.^5^ Three random and same-sized images (300 µm × 300 µm) of alveoli were captured from each lung under a microscope (scale bar = 50 µm). The measurement of MLI was performed by the grid passing through the image. The MLI was obtained from the results of the total length (L) of each line of the grid divided by the number of alveolar intercepts (NAI). The MLI was calculated from the formula MLI = L/NAI. The airspace surface (S) divided by the number of alveoli (NA) was used for the measurement of MAA. The MAA was calculated from the formula MAA = S/NA. Statistical analysis of MLI and MAA was performed using SPSS Statistics for Windows, Version 18.0 (SPSS Inc., Chicago, IL, USA) and data are expressed as mean ± standard deviation. The level of statistical significance was set at *P* < 0.05.

The results are presented in Fig. 1. Compared with the control mice, the other three groups had more pulmonary inflammatory cell infiltration and small airway remodeling. In particular, PM mice exhibited the most severe inflammatory cell infiltration, mucous cell metaplasia, and mucus plugging of the lungs. In contrast, CS and PM–CS mice had scattered and restricted inflammatory cells, mucous cell metaplasia and mucus plugs in some airway lumens (Fig. 1A, B). Muc5ac is an essential biomarker of airway mucus hypersecretion.^6^ IHC revealed that muc5ac was significantly upregulated in the lungs of PM mice and moderately upregulated in the lungs of PM–CS mice. However, upregulation of muc5ac in CS mice was not evident (Fig. 1C).

**Fig. 1:**
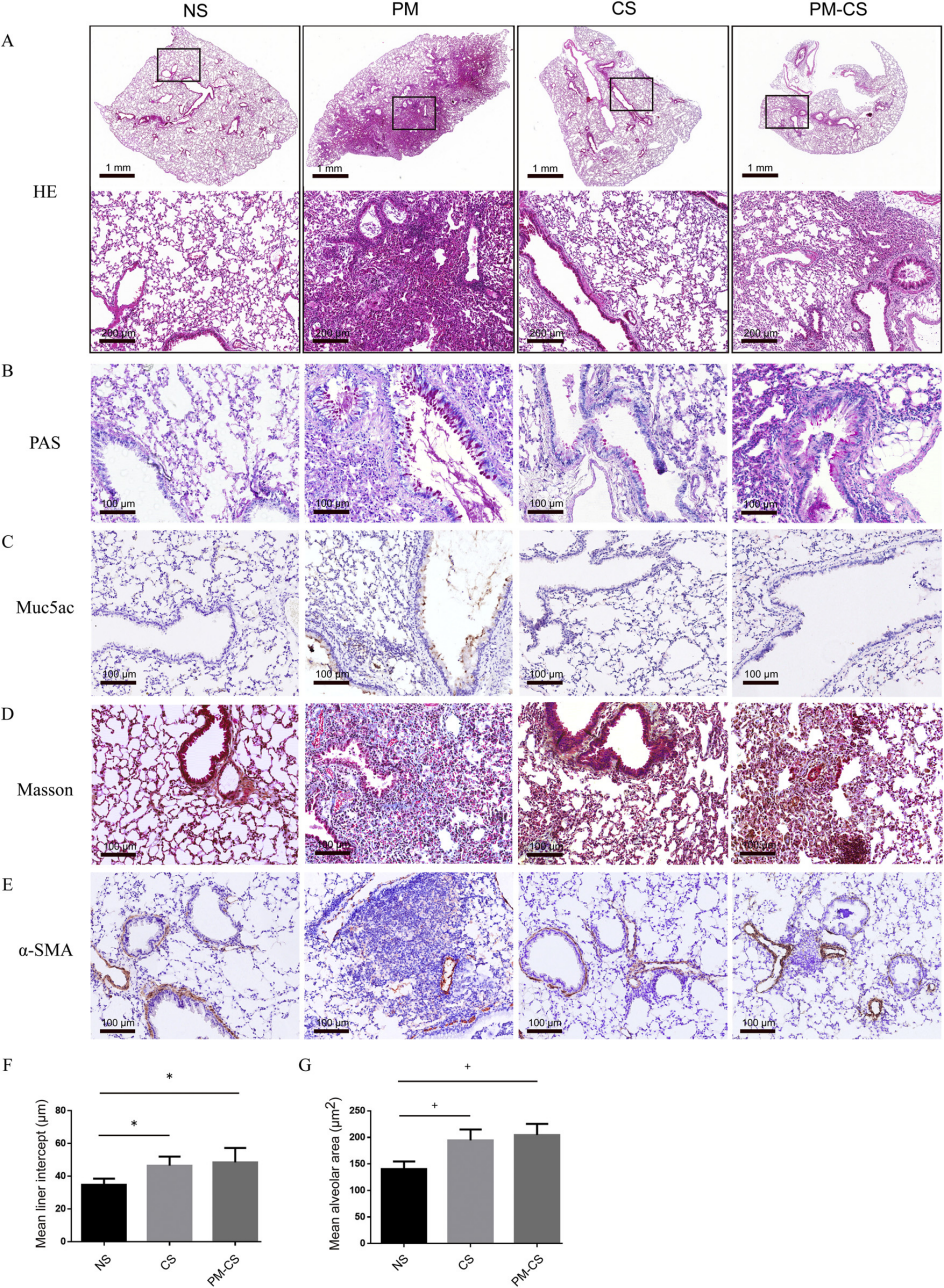
Lung injuries under different exposure conditions. (A) Representative images of HE-stained lung sections from the indicated groups of mice. The box in the upper panel is magnified in the bottom panel. Scale bars represent 1 mm in the upper panel and 200 µm in the bottom panel. (B) Representative images of PAS-stained lung sections from the indicated groups of mice. Mucus plugs in the airway lumen and mucous cell metaplasia were PAS-positive. Scale bar, 100 µm. (C) Representative images of immunohistochemical staining for Muc5ac in lung sections. Scale bar, 100 µm. (D) Representative images of Masson-stained lung sections from the indicated groups of mice. Collagen fibers in the airway and lung parenchyma are stained blue. Scale bar, 100 µm. (E) Representative images of immunohistochemical staining for α-SMA in lung sections. Scale bar, 100 µm. (F–G) Quantification of the mean alveolar area (MAA) and mean linear intercept (MLI). The data are presented as the means ± SDs, *n* = 4. *P < 0.05; ^†^*P* < 0.01. α-SMA: α-smooth muscle actin; CS: Cigarette smoke; HE: Hematoxylin-eosin; Muc5ac: Mucin 5AC; NS: Normal saline; PAS: Periodic acid-Schiff; PM: Particulate matter; SD: Standard deviation.

Next, Masson's trichrome revealed that the PM mice exhibited a marked increase in collagen deposition. Nevertheless, this phenomenon was not obvious in either the CS or PM–CS mice (Fig. 1D). The expression of α-SMA indicates the formation of tissue fibrosis. IHC staining for α-SMA revealed that it was significantly upregulated in the lungs of PM mice and slightly upregulated in the lungs of CS and PM–CS mice (Fig. 1E).

Finally, emphysematous lesions were assessed using the MLI and MAA. The MLI and MAA scores were significantly greater in CS mice (*P* = 0.029 for MLI and *P* = 0.003 for MAA) and PM–CS mice (*P* = 0.014 for MLI and *P* = 0.001 for MAA) than in control mice, suggesting that CS mice and PM–CS mice suffer from emphysematous lesions. However, no significant differences were observed between the PM–CS and CS groups (*P* = 0.651 for MLI and *P* = 0.464 for MAA), despite the slightly higher MLI and MAA scores in the PM–CS group (Fig. 1F–G). MLI and MAA analyses were not performed in PM mice due to the large number of inflammatory cells in the alveolar area, which can cause considerable measurement bias.

Phenotype heterogeneity in COPD patients is the result of endotype heterogeneity. Unfortunately, there is limited research on the associations among the underlying causative mechanisms, endotypes and phenotypes of COPD, especially the interactions among different risk factors. Our pilot findings provide pathological evidence for the phenotypes of single and combined environmental risk factor-induced lung injuries.

On the one hand, we found significant differences in pulmonary pathological characteristics between PM-induced and cigarette smoke-induced pulmonary inflammation. Three-month PM exposure caused pronounced airway inflammation, mucus hypersecretion and airway remodeling of lung tissue. In contrast, during the same duration of exposure to cigarette smoke, only inflammatory cell aggregation, alveolar wall damage, and localized mucous cell metaplasia of epithelial cells were observed in lung. The results highlight the distinct pathological pathways triggered by different environmental exposures, which suggested that the phenotypic manifestations of COPD may vary significantly depending on the predominant environmental risk factor involved. Future researches should focus on elucidating the molecular mechanisms underlying these differences and exploring potential therapeutic targets tailored to specific environmental exposures.

On the other hand, interestingly, no obvious synergistic effects were observed between PM and cigarette smoke. Compared with CS exposure, overlapping exposure to PM and CS aggravated localized mucus secretion and inflammatory cell infiltration. Nevertheless, lung inflammation caused by overlapping exposure was less severe than the damage caused by PM exposure alone. These results indicated that while both PM and CS contribute to lung injury, the specific mechanisms and severity of their combined effects may differ from those of individual exposures.

However, these findings are not consistent with previous studies. A previous study showed that long-term autonomous inhalation of PM2.5 aerosols in mice indicated that the pathological effect of PM2.5 on normal lung tissues was not significant, but there were significant changes in inflammatory factors.^7^ In terms of emphysematous lesions, our results indicated that the combined exposure to PM and CS modestly aggravated the alveolar destruction induced by simple CS, yet no significant difference was observed between the two groups. Gu et al^7^ reported that emphysema was more significant with continuous autonomous inhalation of PM2.5 combined with cigarette smoke exposure than with simple cigarette smoke exposure. The overall difference in the treatment and concentration of PM varied greatly between the two studies, resulting in opposite results.

From the “exposome” perspective, these findings underscore the complexity of environmental interactions in respiratory diseases. The “exposome” encompasses all environmental exposures from conception onwards.^8^ In exposome–disease association, it is increasingly recognized that the combined effects of multiple exposures may not simply be cumulative. Our correspondence primarily showed that the cumulative effect of ambient PM and cigarette smoke was not merely the simple sum of their individual effects. In the exposome of COPD, CS appears to be the primary driver, with PM acting as a secondary contributor. Whether cigarette smoking might predominate in individuals with overlapping exposure cannot yet be explained. The underlying mechanism is not yet clear. Therefore, clinical and experimental studies are needed to determine the phenotypes and endotype mechanisms of overlapping environmental risk factors in COPD patients in the future. This area of research still presents considerable challenges and opportunities for future investigation.

## CRediT authorship contribution statement

**Lifeng Yan:** Writing – original draft, Validation. **Huaqi Guo:** Writing – original draft, Validation. **Juan Fu:** Writing – review & editing, Supervision. **Tianyu Zhou:** Writing – review & editing, Project administration, Conceptualization, Investigation.
